# Process approach as a cognitive biomarker related to gray matter volume in mild cognitive impairment and Alzheimer’s disease

**DOI:** 10.1186/s12883-024-03711-2

**Published:** 2024-06-13

**Authors:** Wenhao Zhu, Xia Zhou, Mengmeng Ren, Wenwen Yin, Yating Tang, Jiabin Yin, Yue Sun, Xiaoqun Zhu, Zhongwu Sun

**Affiliations:** 1 Department of Neurology, the First Affiliated Hospital of Anhui Medical University, 218 Jixi Road, Shushan District, Hefei, 230022 China; 2 Department of Neurology, the Second Affiliated Hospital of Anhui Medical University, Hefei, China

**Keywords:** Process approach, Gray matter volume, Mild cognitive impairment, Alzheimer’s disease, Neuropsychological assessment

## Abstract

**Background:**

Process approach is valuable for memory assessment in Alzheimer’s disease (AD) and mild cognitive impairment (MCI), yet its underlying mechanisms remain elusive. This study aims to synergize the process approach with brain structure analysis to explore both the discriminative capacity and potential mechanisms underlying the process approach.

**Methods:**

37 subjects of MCI, 35 subjects of AD and 38 subjects of healthy control (HC) were included. The process approach in Auditory Verbal Learning Test (AVLT), including discriminability (A’), response bias (B"_D_), semantic clustering (LBC_sem_) and serial clustering (LBC_ser_) was performed. The gray matter volume (GMV) was analyzed by voxel-based morphometry. Receiver operating characteristic (ROC) analysis and partial correlations were conducted to explore the value of the process approach and investigate the relationship between the process approach, traditional indices of AVLT and GMV.

**Results:**

ROC analysis showed the value of A’, B"_D_ and LBC_ser_ in differentiating MCI and AD. Combining AVLT-Immediately Recall (AVLT-IR) and LBC_ser_ showed a higher value in diagnosing MCI. Partial correlations revealed that in the MCI group, A’ and B"_D_ were mainly positively associated with GMV of the hippocampus and temporal lobe.

**Conclusion:**

This study indicated that the process approach is a promising cognitive biomarker to detect MCI and AD.

**Supplementary Information:**

The online version contains supplementary material available at 10.1186/s12883-024-03711-2.

## Introduction

Alzheimer’s disease (AD) typically shows progressive memory impairment and is the leading cause of dementia. Mild cognitive impairment (MCI), as the intermediate stage between normal aging and dementia, is characterized by cognitive decline with preserved independence of function in daily life [1]. A cross-sectional study of dementia indicated that the prevalence in China was estimated to be 3.9% for AD, and 15.5% for MCI [2], making it a crucial public health problem.

Currently, the diagnostic criteria for AD have evolved into the ATN framework defined by biomarkers such as amyloid beta (Aβ) and tau. However, there is no consensus on cut-off values of these biomarkers, and the methods used, such as cerebrospinal fluid examination and positron emission tomography, are invasive and unsuitable for mass screening, limiting the clinical application of the ATN framework [3]. As a result, neuropsychological assessment is still crucial in diagnosis of AD and MCI with non-invasive and easy-to-conduct characteristics. Episodic memory impairment is a prominent feature of patients with MCI and AD [4, 5]. Wordlist memory tests of neuropsychological assessment, such as California Verbal Learning Test-Second Edition (CVLT-II) [6], Hopkins Verbal Learning Test-Revised (HVLT-R) [7] and Auditory Verbal Learning Test (AVLT) [8] are the most commonly used measures of verbal episodic memory in clinical and research settings. Wordlist memory tests typically have 3 procedures: immediate recall, delay recall and recognition. Traditional indices include the number of correct words in immediate recall, delay recall and recognition.

However, with the rising need for more precise and granular measures of cognitive changes, especially in MCI, traditional indices have failed to provide adequate accuracy and information. The process approach has been commonly employed to measure subtle cognitive changes, monitor progressive cognitive decline, and assess treatment effects [9]. It is the learning abilities, strategies, quantification of errors or other aspects in the neuropsychological assessment [10]. The process approach does not lengthen the testing process and typically maintains the standardized administration and psychometric characteristics of the original tests. It is also an important supplement when delay recall scores approach zero (“floor effects”). Thomas et al. added the several indices of process approach to the diagnostic criteria for different stages of subtle cognitive decline (SCD), which improved the predictive accuracy of the progression of cognitive impairment in SCD and was consistent with the results of Aβ and tau [11]. The AVLT-Huashan version [8], a process-oriented task similar to CVLT-II, has been widely used [12, 13].

The process approach includes a series of indices. In this study, we chose common indices as followed: discriminability, response bias, semantic and serial clustering as process approach indices. There were researches about other indices such as learning ratio [14] and serial position effects [15] but not include in our study. The level of performance in yes–no recognition is reflected in the number of correct hits (*H*) and false-positive (*FA*) errors. According to signal detection theory, which provides a general theoretical framework for understanding how variability in-memory representations of target and distractor items interacts with a cognitive decision process [16], *H* and *FA* yield two measures of recognition: discriminability and response bias [17]. Discriminability refers to the ability to distinguish target words from distractor words. Performance in recognition is also influenced by response bias, which is the tendency to favor “yes” or “no” responses (liberal or conservative, respectively), particularly when there is uncertainty about the correct response. On the other hand, neuropsychologists have proposed two common learning strategies for wordlist memory tests: semantic clustering and serial clustering. Semantic clustering describes the phenomenon that individuals reorganized items based on a shared semantic category and then consecutively recalled semantically related words. Serial clustering reflects that participants recall words in the same order in which they were presented [18].

Memory impairment in individuals with MCI and AD is thought to be associated with the atrophy of related gray matter structures. Some studies have reported MCI and AD subjects displayed significant gray matter volume (GMV) atrophy in the medial temporal lobe (MTL), including the hippocampal region and the parahippocampal gyrus, as well as the posterior cingulate gyrus and amygdala compared with age-matched controls [19, 20].

Previous research has indicated that the process approach played an important role in diagnosing and tracking the progression of cognitive impairment. Most researchers found that AD subjects had impaired discriminability and more liberal response bias [21, 22]. Discriminability was helpful in predicting conversion from MCI to AD, and adding discriminability to the delay recall created a better predictive tool [23, 24]. The amnestic-MCI (aMCI) and AD participants displayed worse semantic clustering [25, 26]. Moreover, impaired semantic clustering in aMCI subjects was attributed to MTL atrophy [27]. Researchers indicated that semantic encoding might related to brain networks including MTL. In comparison, few studies have focused on serial clustering. A study indicated that serial clustering had no significant difference in subjects with the apolipoprotein E (APOE) ε4 allele, a major risk factor for AD [28]. A recent study investigated the performance of electronic version of HVLT-R in AD and aMCI, and found that compared with healthy control (HC) subjects, semantic clustering declined in aMCI subjects while serial clustering was preserved; both semantic and serial clustering declined in the AD stage [29].

Many researchers paid attention to the process approach in neuropsychology in MCI or AD, while MRI-related studies were not universal. In addition, serial clustering and response bias have not been typically realized. Because of the differences in procedures, content and length of wordlist memory tests, it remains unclear whether the process approach above in AVLT correlates with GMV in MCI and AD. Therefore, the following hypotheses were proposed in this study: (1) one or more indices of the process approach show significant differences between MCI, AD and HC subjects and are promising cognitive biomarkers in MCI and AD; (2) process approach is correlated with the GMV of specific clusters.

## Methods

### Subjects

A total of 110 participants, including 37 subjects of MCI, 35 subjects of AD and 38 subjects of HC, were recruited from the First Affiliated Hospital of Anhui Medical University between September 2020 and June 2023. This study was approved by the Institution Ethics Committee of the First Affiliated Hospital of Anhui Medical University (PJ 2023-12-39). Written informed consent was obtained from all participants or their legal guardians following the Declaration of Helsinki after a full explanation of the procedure. All participants underwent collection of demographic data, detailed medical history, physical examination, neuropsychological assessment, laboratory tests and brain MRI scan.

The diagnosis of MCI and probable AD met the criteria of the National Institute of Aging and Alzheimer’s Association (NIA-AA) [5, 30]. All participants were 55 ∼ 80 years old and had elementary education or above (≥ 5 years of education).

The inclusion criteria for the MCI group were as follows: (1) subjective cognitive complaints by participants or informants; (2) Mini-Mental State Examination (MMSE) [31] score < 25 for 5 ∼ 6 years of education; < 28 for individuals with 7 or more years of education according to a population-based study of MMSE in elderly Chinese [32]; (3) preserved activities of daily living, Activities of Daily Living (ADL) [33] score ≤ 22; (4) absence of dementia, according to the Diagnostic and Statistical Manual of Mental Disorders, Fourth Edition, Text Revised (DSM-IV-TR) [34].

The inclusion criteria for the AD group were as follows: (1) gradual onset of cognitively impaired symptoms for over 3 months and affected instrumental and essential daily function by participants or informants; (2) MMSE score < 20 for 5 ∼ 6 years of education; < 24 for individuals with 7 or more years of education.

The inclusion criteria for the HC group were as follows: (1) no cognitive complaints and having the average function in activities of daily living; (2) MMSE score ≥ 25 for 5 ∼ 6 years of education; ≥ 28 for individuals with 7 or more years of education; (3) Clinical Dementia Rating (CDR) [35] = 0.

The exclusion criteria were as follows: (1) history of stroke, brain tumor, traumatic brain injury, epilepsy, psychiatric disease, cognitive impairment with other core clinical features such as dementia with Lewy bodies, frontotemporal dementia or other neurological disease; (2) presence of multiple lacunar cerebral infarctions or severe white matter hyperintensity (Fazekas grade [36] ≥ 2); (3) use of medication that could have a substantial effect on cognition, alcohol or substance abuse; (4) severe heart, liver or kidney diseases, thyroid disease or tumor; (5) depression with Hamilton Depression Rating Scale (HAMD) [37] score > 7; (6) severe hearing or visual impairment; (7) unable to cooperate with MRI scan; (8) severe dementia with CDR ≥ 2.

### Neuropsychological assessment

Neuropsychological assessment of all participants was performed by trained neuropsychological technicians within 1 week of the MRI scan. All participants were assessed using the MMSE, Montreal Cognitive Assessment (MoCA) [38], AVLT-Huashan version, HAMD, CDR, and ADL.

The AVLT consists of 3 learning trials that included a list of 12 two-characters words divided into three semantic categories (flowers, occupations and apparels) followed by delay recall and recognition. After reading the 12 words, participants were asked to recall as many words as they could remember immediately. The words of 3 trials were read in the same order. The correct words of 3 trials were summed for the Immediately Recall (AVLT-IR, range = 0 ∼ 36). After a 5-min and a 20-min interval in which non-verbal tests were performed, participants were asked to recall the words freely, namely short-term delay recall (AVLT-SR, range = 0 ∼ 12) and long-term delay recall (AVLT-LR, range = 0 ∼ 12). After the long-term delay recall, a yes–no recognition task was administered, including 12 target and 12 distractor words, and the correct words were summed (AVLT-REC, range = 0 ∼ 24). AVLT-IR, AVLT-SR, AVLT-LR and AVLT-REC are traditional indices of AVLT.

There were different ways of calculating the process approach. We used the formulas in **supplemental materials** to measure the process approach of discriminability (A’), response bias (B"_D_), semantic clustering (LBC_sem_) and serial clustering (LBC_ser_).

### MRI data acquisition

MRI scans were obtained using a 3.0-Tesla MR system (Discovery MR750w, General Electric, Milwaukee, WI, USA) with a 24-channel head coil. Earplugs were used to reduce noise, and tight but comfortable foam padding was used to minimize head motion. High-resolution 3D T1-weighted structural images were acquired by employing a brain volume (BRAVO) sequence with the following parameters: repetition time (TR) = 8.5 ms; echo time (TE) = 3.2 ms; inversion time (TI) = 450 ms; flip angle (FA) = 12°; field of view (FOV) = 256 mm×256 mm; matrix size = 256 × 256; slice thickness = 1 mm, without gap; 188 sagittal slices; and acquisition time = 296 s. T2 FLAIR images were acquired with the following parameters: TR = 9,000 ms, TE = 119.84 ms, FA = 160°, FOV = 225 mm×225 mm, matrix size = 512 × 512, 19 contiguous slices of 7.0 mm thickness, and scan time = 118 s. All MRI images were visually inspected without inadequate quality.

### VBM analysis

The Computational Anatomy Toolbox 12 software (CAT12, https://www.neuro.uni-jena.de/cat) was used to calculate GMV based on Statistical Parametric Mapping software (SPM8, https://www.fil.ion.ucl.ac.uk/spm). Structural MRI images were segmented into gray matter, white matter (WM) and cerebrospinal fluid (CSF) using a standard segmentation model. After the initial affine registration of the gray matter concentration was mapped into Montreal Neurological Institute (MNI) space, the gray matter concentration images were nonlinearly warped using the Diffeomorphic Anatomical Registration Through Exponentiated Lie algebra (DARTEL) technique and then resampled to a voxel size of 1.5 mm×1.5 mm×1.5 mm. GMV was obtained by multiplying the gray matter concentration map by the non-linear determinants derived from the spatial normalization step. Finally, the GMV images were smoothed using a Gaussian kernel of 8 mm×8 mm×8 mm full-width at half maximum.

A multiple regression model in SPM8 was used to identify brain structural changes between different groups, controlling for age, gender, education, and total intracranial volume (TIV) as covariates. TIV was calculated by summing the total GMV, WMV, and CSF volumes. Multiple comparison correction was performed using the voxel-level family-wise error (FWE) method. Resting-State fMRI Data Analysis Toolkit plus (RESTplus, https://www.restfmri.net/forum/restplus) software was used to extract the GMV of structurally changed brain regions for further analysis.

### Statistical analyses

Statistical Product Service Solutions (SPSS, v.23.0), GraphPad Prism (v.8.0.2), R software (v.4.2.2) package ggplot2 (v.3.4.2) and Medicine Calculator (MedCalc, v.19.7.2) were used for statistical analysis and visualization. Normally distributed continuous variables were expressed as mean ± standard deviation (SD), and comparisons among the three groups were analyzed by one-way analysis of variance (ANOVA). Bonferroni correction was used for *post hoc* group comparisons. Non-normally distributed numerical variables were expressed as medians (P_25_, P_75_), and comparisons among the three groups were performed using the Kruskal–Wallis test. Categorical variables were expressed as n (%), and Pearson’s chi-squared analysis was used to compare the three groups. Statistical significance was defined as two-tailed *p* < 0.05.

Receiver operating characteristic (ROC) analysis was performed, and the maximum area under the curve (AUC) and confidence interval of 95% (95%CI) were calculated. Binary logistic regression was used for ROC analysis of the combined index. Comparisons of AUCs were performed by DeLong’s test [39].

Partial correlations were performed between the process approach and traditional indices of AVLT, the process approach and GMV among three groups by Pearson’s correlation, controlling for age, gender and education as covariates. Partial correlation coefficients (*pr*) were calculated. False Discovery Rate (FDR) correction was used for adjusted *p* values.

## Results

### Demographic and neuropsychological assessment results

The demographic and neuropsychological assessment results are shown in Table 1. There were no significant differences in age, gender or education among the three groups (*p* > 0.05). The MCI group exhibited significantly lower MMSE (*p* < 0.001), MoCA (*p* = 0.001), CDR (*p* = 0.001), AVLT-IR (*p* < 0.001), AVLT-SR (*p* = 0.032) and AVLT-REC (*p* = 0.032) than the HC group. The AD group exhibited significantly lower MMSE, MoCA, CDR, ADL, AVLT-IR, AVLT-SR, AVLT-LR and AVLT-REC than both the MCI and HC groups (AD versus MCI, AD versus HC, *p* < 0.001).

**Table 1:** Demographic and neuropsychological assessment of all participants

	HC (*n* = 38)	MCI (*n* = 37)	AD (*n* = 35)	χ^2^, F, H	*p*
Age, y	64.66 ± 6.54	66.19 ± 5.73	67.63 ± 8.15	1.714^a^	0.185
Gender, female	17 (45%)	15 (41%)	19 (54%)	1.428^b^	0.490
Education, y	10.50 ± 3.20	10.51 ± 2.74	10.03 ± 2.47	0.341^a^	0.712
MMSE	29 (28, 29)	27 (26, 27) ^†^	19 (16, 22) ^†‡^	92.039^c^	**< 0.001**
MoCA	25.5 (24, 27)	22 (20, 23.5) ^†^	13 (10, 16) ^†‡^	78.918^c^	**< 0.001**
CDR	0 (0, 0)	0.5 (0.5, 0.5) ^†^	1 (0.5, 1) ^†‡^	87.139^c^	**< 0.001**
ADL	20 (20, 20)	20 (20, 20)	25 (23, 27) ^†‡^	86.098^c^	**< 0.001**
AVLT-IR	17.63 ± 3.37	14.30 ± 3.94^†^	8.57 ± 3.42^†‡^	58.986^a^	**< 0.001**
AVLT-SR	6.5 (5, 7.25)	5 (3, 6) ^†^	1 (0, 2) ^†‡^	64.162^c^	**< 0.001**
AVLT-LR	5 (4, 8)	4 (2, 6.5)	0 (0, 0) ^†‡^	66.275^c^	**< 0.001**
AVLT-REC	22 (20, 23)	20 (17, 22) ^†^	15 (12, 17) ^†‡^	51.106^c^	**< 0.001**

HC, health control; MCI, mild cognitive impairment; AD, Alzheimer’s disease; MMSE, Mini-Mental State Examination; MoCA, Montreal Cognitive Assessment; CDR, Clinical Dementia Rating; ADL, Activities of Daily Living; AVLT-IR, Auditory Verbal Learning Test-Immediately Recall; AVLT-SR, Auditory Verbal Learning Test-short-term delay recall; AVLT-LR, Auditory Verbal Learning Test-long-term delay recall; AVLT-REC, Auditory Verbal Learning Test-recognition. Data are given as n (%), mean ± SD, median (P_25_, P_75_), ^a^ANOVA test, ^b^chi-square test, ^c^Kruskal-Wallis test, ^†^compared to HC group *p* < 0.05, ^‡^compared to MCI group *p* < 0.05

**Fig. 1:**
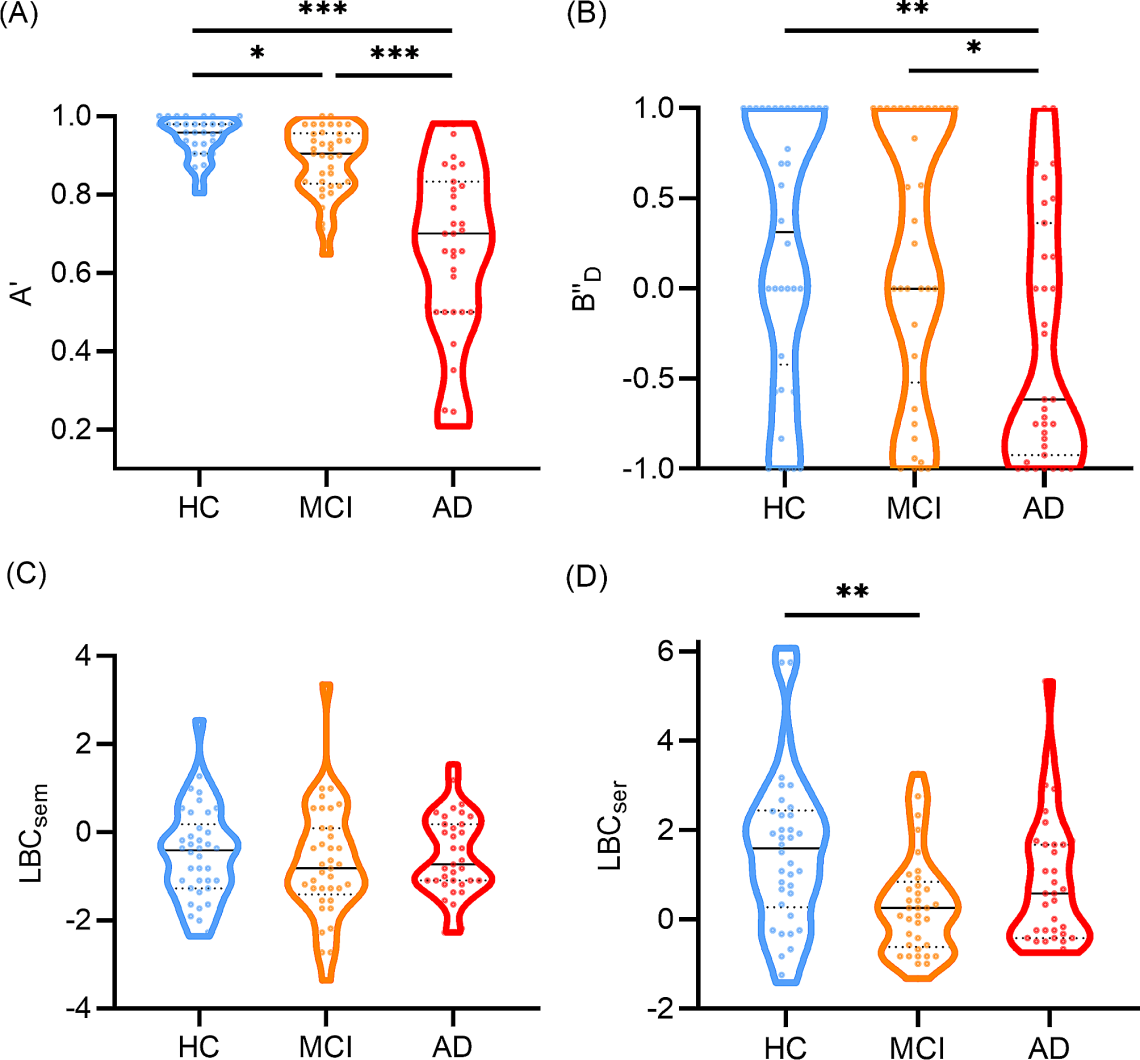
Comparisons of process approach between the HC, MCI and AD groups. A’ is presented as median (P25, P75); B"_D_, LBC_sem_ and LBC_ser_ are presented as mean ± standard deviation. A’, discriminability; B"_D_, response bias; LBC_sem_, ListBased Clustering Index of semantic; LBC_ser_, ListBased Clustering Index of serial; HC, health control; MCI, mild cognitive impairment; AD, Alzheimer’s disease. *p < 0.05, **p < 0.01, ***p < 0.001

Comparisons of the process approach among the three groups are shown in Fig. 1. The MCI group exhibited significantly lower A’ than the HC group, and the AD group exhibited significantly lower A’ than the MCI and HC groups (*H* = 43.954, *p* < 0.001; AD versus MCI, AD versus HC, *p* < 0.001; MCI versus HC, *p* = 0.038, Fig. 1A). The AD group exhibited significantly lower B"_D_ than the MCI and HC groups (*F* = 5.662, *p* = 0.005; AD versus MCI, *p* = 0.023; AD versus HC, *p* = 0.007, Fig. 1B). There was no significant difference in LBC_sem_ among the three groups (*F* = 0.409, *p* = 0.665, Fig. 1C). The means of the three groups were all below zero. The MCI group exhibited significantly lower LBC_ser_ than the HC group. (*F* = 6.625, *p* = 0.002; MCI versus HC, *p* = 0.001, Fig. 1D).

### Correlations of the process approach and traditional indices of AVLT

**Fig. 2:**
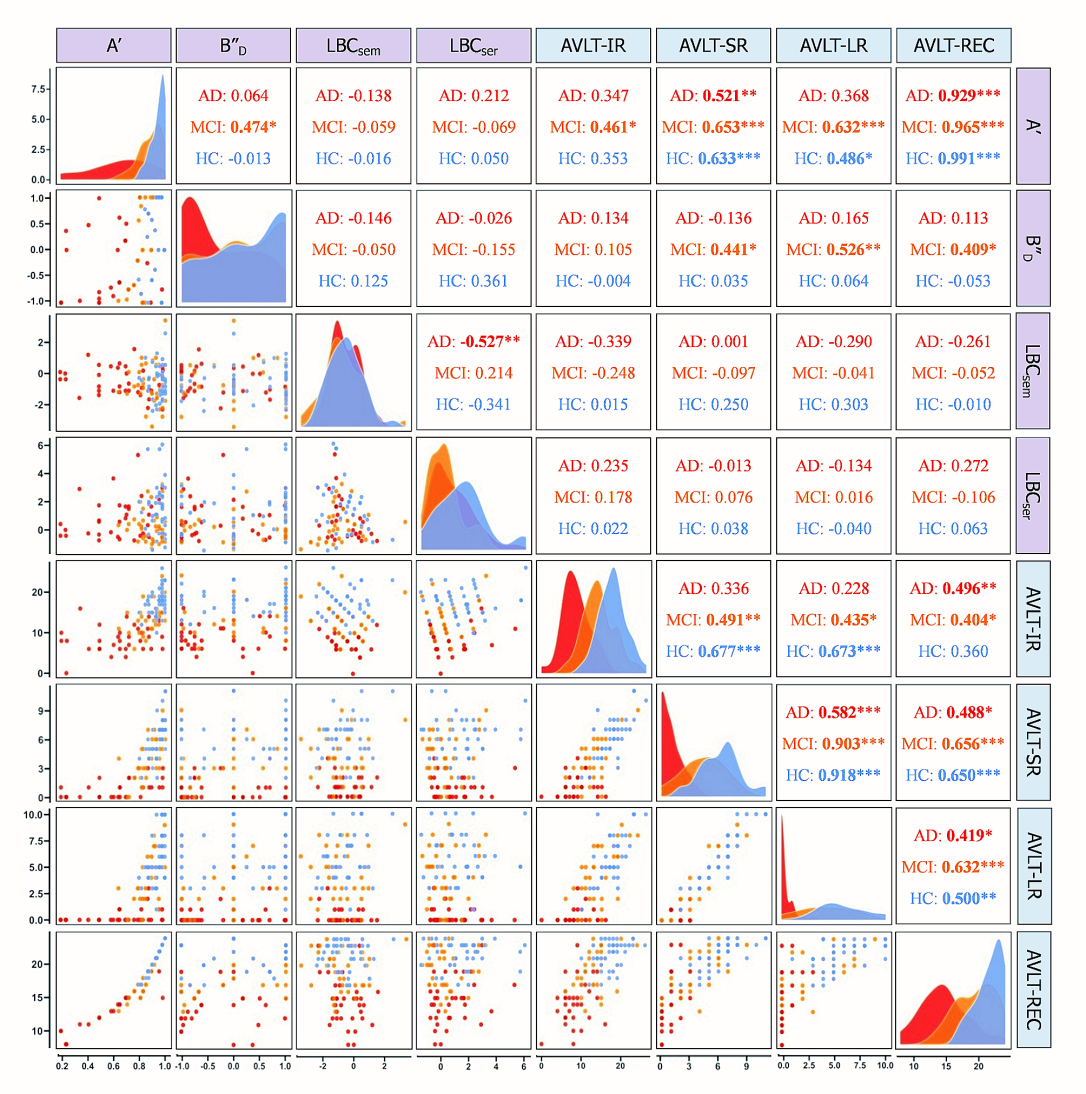
Partial correlations between the process approach and traditional indices of AVLT by Pearson’s correlation after controlling age, gender and education as covariates. Three partial correlation coefficients are shown in the upper right triangle area for every correlation between two indices (one for the AD, MCI and HC groups). FDR (false discovery rate) correction of *p < 0.05, **p < 0.01, ***p < 0.001. Scatter plots for each correlation are shown in the sitting triangle area. The nuclear density curves of each index are displayed in the diagonal cells, where red represents the AD group, orange represents the MCI group, and blue represents the HC group. A’, discriminability; B"_D_, response bias; LBC_sem_, ListBased Clustering Index of semantic; LBC_ser_, ListBased Clustering Index of serial; AVLT-IR, Auditory Verbal Learning Test-Immediately Recall; AVLT-SR, Auditory Verbal Learning Test-short-term delay recall; AVLT-LR, Auditory Verbal Learning Test-long-term delay recall; AVLT-REC, Auditory Verbal Learning Test-recognition; AD, Alzheimer’s disease; MCI, mild cognitive impairment; HC, health control

Partial correlations were performed between the process approach and traditional indices of AVLT among three groups, controlling for age, gender and education as covariates (Fig. 2). It showed that A’ was positively associated with all four traditional indices, especially strongly associated with AVLT-REC (*pr* = 0.461 ∼ 0.991, *p* < 0.05). B"_D_ was positively associated with AVLT-SR, AVLT-LR and AVLT-REC in the MCI group (*pr* = 0.409 ∼ 0.526, *p* < 0.05). There was no significant difference between LBC_sem_, LBC_ser_ and traditional indices. In addition, LBC_sem_ was negatively associated with LBC_ser_ in the AD group (*pr* = − 0.527, *p* < 0.01), whereas no significant results were found in the MCI and HC groups.

### ROC analysis of process approach and traditional indices of AVLT

**Fig. 3:**
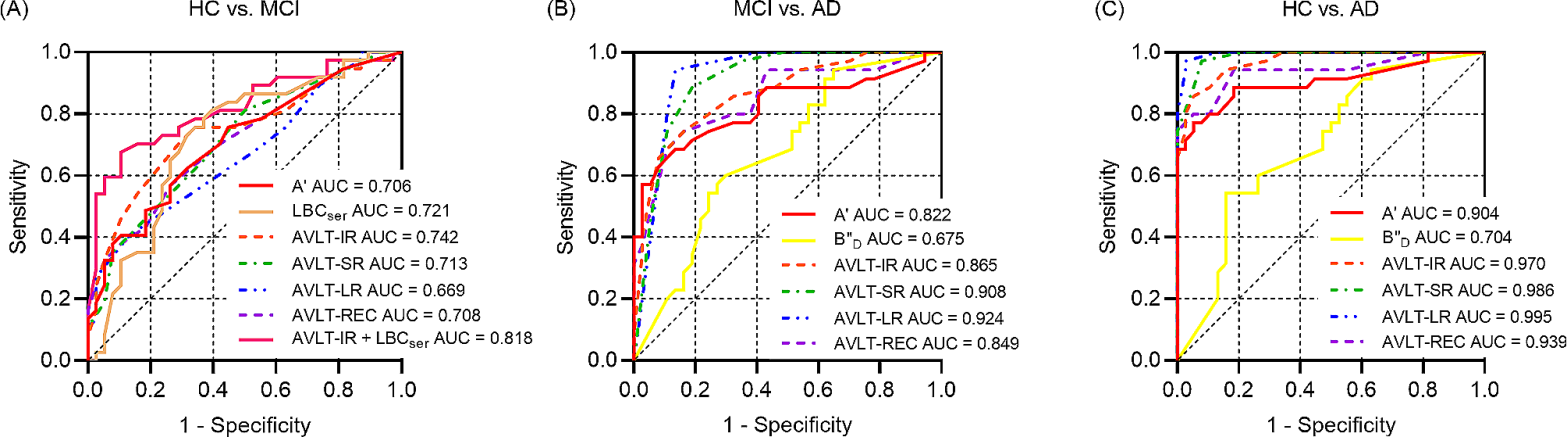
Receiver operating characteristic curves of a process approach and traditional indices of AVLT between the HC, MCI and AD groups. AUC, area under the curve; A’, discriminability; B"_D_, response bias; LBC_ser_, ListBased Clustering Index of serial; AVLT-IR, Auditory Verbal Learning Test-Immediately Recall; AVLT-SR, Auditory Verbal Learning Test-short-term delay recall; AVLT-LR, Auditory Verbal Learning Test-long-term delay recall; AVLT-REC, Auditory Verbal Learning Test-recognition; HC, health control; MCI, mild cognitive impairment; AD, Alzheimer’s disease; vs, versus

Considering the above findings, we speculated that A’, B"_D_ and LBC_ser_ might be potential cognitive markers of MCI and AD. We further determined the diagnostic value of A’, B"_D_ and LBC_ser_ by constructing the ROC analysis (Fig. 3 and Supplemental Table S1, S2). To discriminate between HC and MCI groups, the AUC of A’ was 0.708 (95%CI: 0.592 ∼ 0.807) when the optimal cut-off value was 0.955, and the sensitivity and specificity were 0.757/0.553. The AUC of LBC_ser_ was 0.721 (95%CI: 0.605 ∼ 0.818) when the optimal cut-off value was 1.000, and the sensitivity and specificity were 0.811/0.605. Considering the maximum AUC, we combined process approach and traditional indices as new indices and compared with traditional indices only. We found the AUC of combined index (LBC_ser_ + AVLT-IR) was 0.818 (95%CI: 0.720 ∼ 0.916) when the sensitivity and specificity were 0.676/0.894. There was a significant difference in AUC only between (LBC_ser_ + AVLT-IR) and AVLT-IR (*p* = 0.036) (Fig. 3A).

To discriminate between MCI and AD groups, the AUC of A’ was 0.822 (95%CI: 0.714 ∼ 0.902) when the optimal cut-off value was 0.795, and the sensitivity and specificity were 0.686/0.865. The AUC of B"_D_ was 0.675 (95%CI: 0.555 ∼ 0.781) when the optimal cut-off value was − 0.200, and the sensitivity and specificity were 0.600/0.703. We combined A’ with each traditional index, and Delong’s test showed no statistical significance between these AUCs (*p* > 0.05) (Fig. 3B).

Furthermore, to discriminate between HC and AD groups, the AUC of A’ was 0.905 (95%CI: 0.813 ∼ 0.961) when the optimal cut-off value was 0.833, and the sensitivity and specificity were 0.771/0.947. The AUC of B"_D_ was 0.704 (95%CI: 0.585 ∼ 0.805) when the optimal cut-off value was − 0.615, and the sensitivity and specificity were 0.543/0.842. We also combined A’ with each traditional index and found no statistical significance between these AUCs (*p* > 0.05) (Fig. 3C).

### GMV of the three groups

**Fig. 4:**
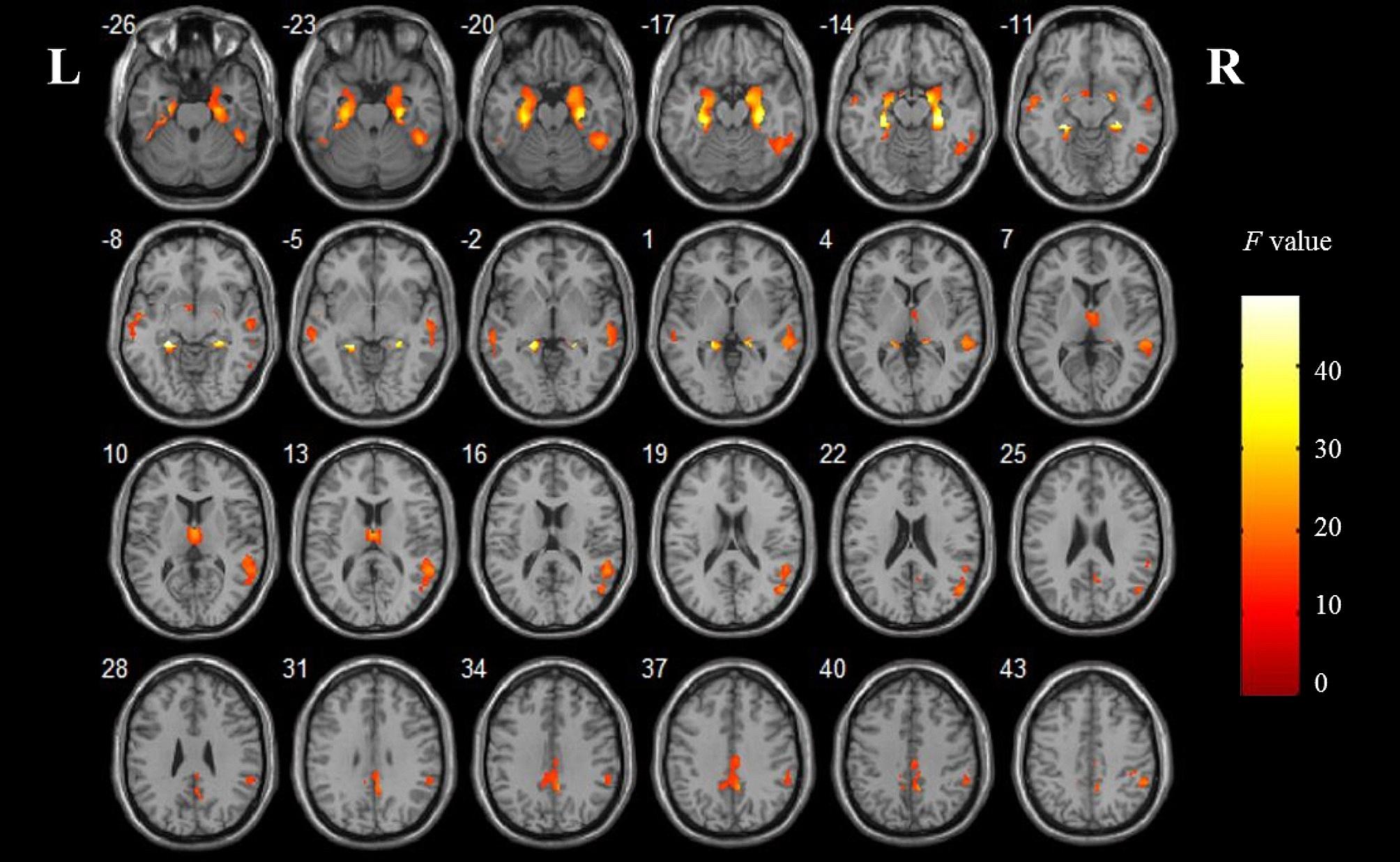
Comparisons of gray matter volume in HC, MCI and AD groups after controlling age, gender, education and TIV as covariates. Significant brain gray matter atrophy was in the right middle temporal gyrus, right hippocampus, left parahippocampal gyrus, right middle cingulate gyrus, right inferior temporal gyrus, right thalamus and left middle temporal gyrus. L, left; R, right

As shown in Fig. 4, after controlling for age, gender, education and TIV as covariates, the comparisons involving three groups revealed a significant effect of GMV on the right middle temporal gyrus (MTG.R), right hippocampus (HIP.R), left parahippocampal gyrus (PHG.L), right middle cingulate gyrus (MCG.R), right inferior temporal gyrus (ITG.R), right thalamus (THA.R) and left middle temporal gyrus (MTG.L) (*p* < 0.05, voxel-level FWE corrected). Intergroup analysis showed a significant diagnostic effect (AD < MCI) and (AD < HC) on the same clusters above. In addition, there was a significant effect (MCI < HC) on the MTG.R. Cluster size and peak point coordinate are shown in Supplemental Table S3.

### Correlations of the process approach and GMV

**Fig. 5:**
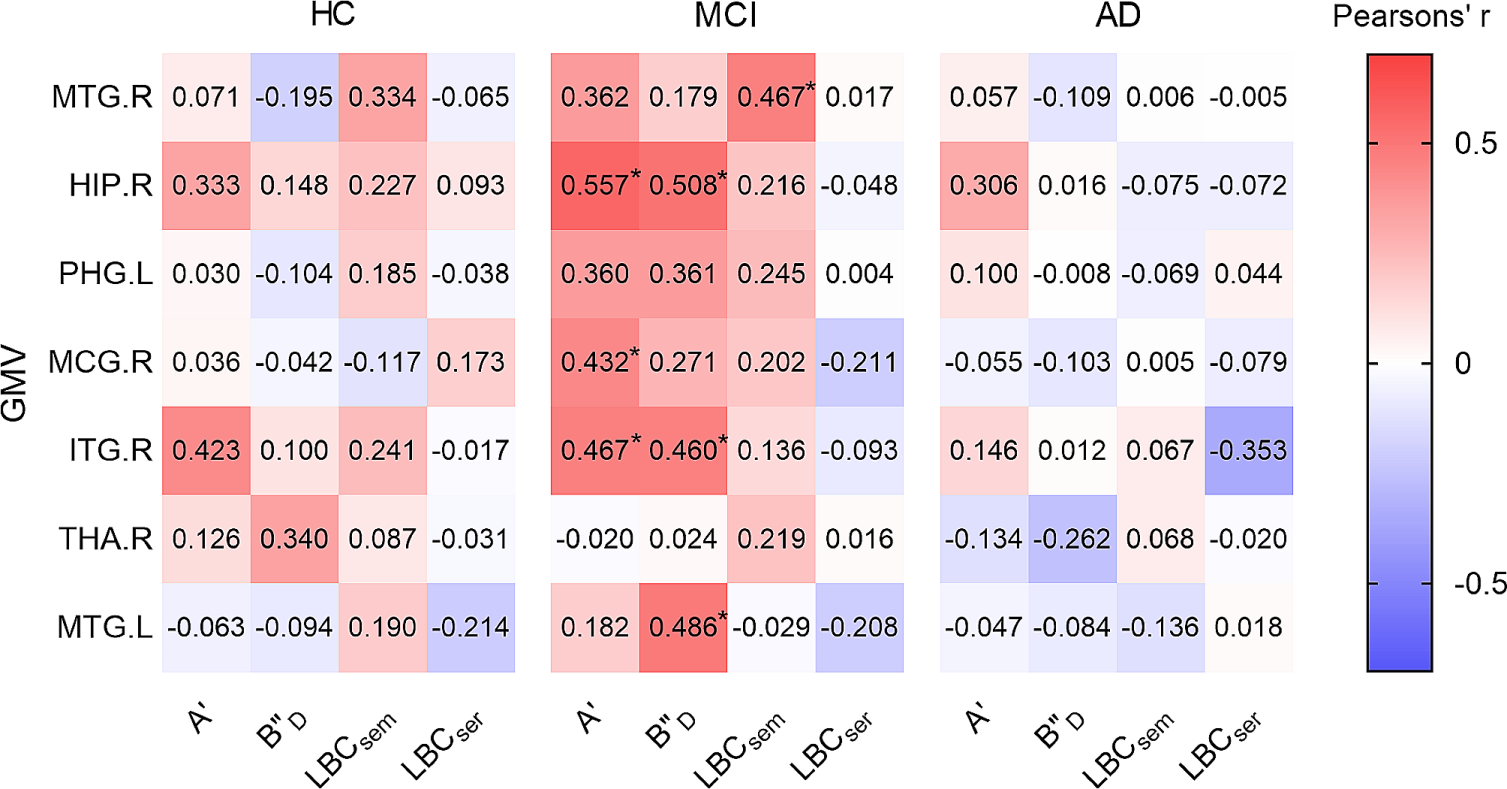
Heat plot of partial correlations between process approach and gray matter volume by Pearson’s correlation after controlling age, gender and education as covariates. Partial correlation coefficients are shown. FDR (false discovery rate) correction of *p < 0.05. A’, discriminability; B"_D_, response bias; LBC_sem_, ListBased Clustering Index of semantic; LBC_ser_, ListBased Clustering Index of the serial; HC, health control; MCI, mild cognitive impairment; AD, Alzheimer’s disease; MTG.R, right middle temporal gyrus; HIP.R, right hippocampus; PHG.L, left parahippocampal gyrus; MCG.R, right middle cingulate gyrus; ITG.R, right inferior temporal gyrus; THA.R, right thalamus; MTG.L, left middle temporal gyrus

Partial correlations were performed between the process approach and GMV among three groups, controlling for age, gender and education as covariates (Fig. 5). We found that in the MCI group, A’ was positively associated with GMV of HIP.R (*pr* = 0.557, 95%CI: 0.148 ∼ 0.791, *p* < 0.05), MCG.R (*pr* = 0.432, 95%CI: 0.055 ∼ 0.683, *p* < 0.05) and ITG.R (*pr* = 0.467, 95%CI: 0.085 ∼ 0.714, *p* < 0.05). B"_D_ was positively associated with GMV of HIP.R (*pr* = 0.508, 95%CI: 0.240 ∼ 0.711, *p* < 0.05), ITG.R (*pr* = 0.460, 95%CI: 0.164 ∼ 0.655, *p* < 0.05) and MTG.L (*pr* = 0.486, 95%CI: 0.210 ∼ 0.678, *p* < 0.05). In addition, LBC_sem_ was positively associated with GMV of MTG.R (*pr* = 0.467, 95%CI: 0.201 ∼ 0.671, *p* < 0.05) in the MCI group. No significant difference of correlation was found between LBC_ser_ and GMV among the three groups (*p* > 0.05).

## Discussion

Considering that the process approach in the Chinese version has not been widely analyzed, this study explored the process approach of the AVLT-Huashan version, and its relationship with GMV in MCI and AD subjects. We found that discriminability, response bias and serial clustering were promising cognitive biomarkers. Especially combining AVLT-IR and LBC_ser_ showed a higher value for early diagnosis of MCI. Neuroimaging studies illustrated the correlations between discriminability, response bias, semantic clustering and GMV of clusters including the hippocampus, temporal gyrus and cingulate gyrus in the MCI group.

According to the current study, discriminability was positively associated with traditional indices of AVLT, especially AVLT-REC. Discriminability and AVLT-REC are indices that depend on the performance of recognition. This task is relatively easier for HC and MCI participants than delay recall, and they usually got nearly full marks as “ceiling effects”. When participants entered the stage of AD, the memory of both recall and recognition declined, leading to a higher value of discriminability in detecting AD. Delay recall showed the optimal identified value among all indices in many studies and was related to cortex-hippocampus network [40, 41]. Our study supported these conclusions and supposed that discriminability was also helpful in discriminating MCI and AD.

Combined with MRI imaging, Wolk et al. found that only the hippocampus was correlated with delay recall performance; discriminability was also correlated with the hippocampus in terms of cortical thickness in AD [42]. In the current study, we found that in the MCI group, discriminability was positively associated with GMV of the hippocampus, middle cingulate gyrus and inferior temporal gyrus. The hippocampus, part of MTL, is mainly concerned with memory, and operates with the neocortex to establish and maintain long-term memory [43, 44]. Furthermore, longitudinal studies have found that MTL atrophy was a good predictor of individuals with MCI who subsequently developed AD [45]. The cingulate gyrus is not thought to be involved in typical memory functions and has reciprocal connections with other memory areas [46]. As the middle cingulate gyrus part of Papez’s circuit, its hypometabolism was correlated to cingulate bundle disruption and then the hippocampal atrophy [47]. We speculated that the middle cingulate gyrus has an intermediate function in memory processing by linking traditional memory regions. The inferior temporal gyrus is associated with global cognitive function [48] and plays an essential role in verbal fluency [49]. In another VBM analysis, MCI and AD subjects exhibited a characteristic pattern of atrophy permitting the differentiation of several stages, first on the MTL, further progressed to the middle and posterior temporal gyrus, even the parietal lobe, then widespread with more severe involvement of the temporoparietal association cortices and the frontal lobe [50]. This pattern of progression fits well with the Braak and Braak neurofibrillary pathological staging scheme in AD [51]. We supposed that discriminability supported memory accuracy and is associated with inevitable brain atrophy.

Response bias is another index calculated in recognition. We found that the AD group exhibited significantly lower (more liberal) response bias than the MCI and HC groups which was consistent with previous studies [21, 22]. However, the results of MCI remain unclear. Russo et al. suggested that MCI subjects had a liberal response bias similar to AD [21], while we did not get significant results on MCI compared to the HC group. According to the distribution-free model, the response bias ranges from − 1 to 1. When the participants had wrong responses, it will have extreme value while *H* is one or *FA* is zero, especially for MCI and HC subjects. Several studies have suggested that the liberal response bias was related to frontal lobe dysfunction [52]. However, we found that response bias was positively associated with GMV of the hippocampus, inferior and middle temporal gyrus in the MCI group, which correlate with memory function. Also, response bias was positively associated with some traditional indices of AVLT in the MCI group. We supposed that response bias was another index reflecting memory function, distinct from discriminability.

Semantic and serial clustering are the two main learning strategies of AVLT. There was no significant difference in semantic clustering among the three groups. Besides, the means of the three groups were all below expectancy. This was inconsistent with previous studies, which suggested that semantic clustering was an effective learning strategy which could identify cognitive impairment, especially in the MCI stage [27, 53]. We administered the probable reasons below. First, several studies showed that the wordlist learning test was positively associated with education [54, 55], and the words in AVLT are written language and lack context. It might be difficult for illiterates or participants with low education to understand and remember. Therefore, we excluded illiterates and participants with less than five years of education, but the mean education of all participants was about ten years, which was lower than other studies. Even the participants in the HC group might have relatively inadequate ability to obtain semantic categories. Second, compared with other studies of semantic clustering in the Chinese population using primary clustering data [8], our calculation methods came from CVLT-II and considered expectancy to correct chance recall. The expectancy of semantic clustering would be higher when participants recalled more words. It might narrow the gaps between HC and MCI/AD subjects. Besides, a study in the Chinese population used HVLT-R and found that aMCI subjects showed significantly lower semantic clustering [27]. These seemingly conflicting results were not actually contradictory. HVLT-R Chinese version has 12 words divided into three semantic categories similar to AVLT. However, three words in one category “jewels” had the same character “shi” (“stone” in English). Researchers did not mention the details of each semantic category, but we suspected that it might be easier to recall these jewels continuously and increase semantic clustering.

We found that the MCI group exhibited significantly less serial clustering than the HC group. Neuropsychologists have suggested that semantic clustering is an effective learning strategy in which subjects actively reorganize items based on a shared semantic feature; in contrast, serial clustering is an automatic and more passive strategy [6]. Besides, several studies found no significant difference between the HC group and MCI/AD groups in serial clustering [27, 56]. We supposed the possible explanations. First, there are few phoneme-semantics conversion rules and lots of homophonic words in Chinese compared to alphabetic language. Chinese participants could recall the words in the same order even if they did not understand the meaning of the words. In addition, semantic clustering was negatively associated with serial clustering in the AD group in partial correlations. It appears to be a trade-off between clustering strategies such that the less one uses a semantic strategy, the more one uses a serial strategy. When semantic clustering becomes challenging and limits working memory capacity to hold categorize information semantically, some participants might automatically switch to serial clustering [57]. ROC analysis showed that there was a significant difference in AUC between (AVLT-IR + LBC_ser_) and AVLT-IR in the MCI group, making serial clustering not only another sensitive cognitive biomarker for identifying MCI, but also increasing the diagnostic value when combined with traditional index. Besides, contrary to our intuitive thought that learning strategies would decrease with the aggravation of cognitive impairment, we found that the mean serial clustering of the AD group was intermediate between the MCI and HC groups. We further reviewed the original test data and detected that AD subjects tended to recall the first two words and/or the last two words in order in all three learning trails and increased the serial clustering even if they recall few words overall. This was partly due to the serial position effect, in which more words are recalled at the beginning and end than in the middle of the list and is represented by a U-shape learning curve [58]. Also, AD’s lacks of set-shifting ability and mental flexibility was a possible reason [59].

There was almost no significant difference when comparing the association between clustering and GMV, except that semantic clustering was positively associated with GMV of middle temporal gyrus in the MCI group. The null results warrant some discussions. First, for quite a few AD subjects, AVLT-IR ranged from 0 to 3. The clustering would have an all-or-none phenomenon with considerable variability. Besides, individuals varied in the strategies they used to learn lists of words. Examples of common learning strategies include semantic clustering, serial clustering, the position of words on the list (i.e., primacy and recency effects) and idiosyncratic strategies such as recalling pairs of words consecutively based on their functional or phonemic properties (i.e., subjective clustering) [60]. We supposed that semantic and serial clustering were only parts of strategies and there were other learning methods been used. It is very likely that a certain proportion of HC participants might not fully utilize semantic clustering as a learning strategy. And MCI or AD subjects still utilized semantic clustering to some extent [26]. Furthermore, the neuroimaging markers might not have sufficient variability in the AD group due to extensive atrophy.

Specific details had to be mentioned. The process approach in the current study was a valuable tool mainly for providing more information about memory profiles, such as response bias and learning strategies. We emphasized that combining the process approach and traditional indices might be more useful in clinical applications. Also, improvements in neuropsychological assessment can sometimes occur with the development of new theories and formulas and directly lead to different results [61]. Future research should strive to develop newer and better formulas. Last but not least, the length of the wordlist, different characters and test procedures may impact contest procedures and the final results [57]. Accordingly, conclusions from different wordlist learning tests cannot be directly compared.

Nevertheless, our study had several limitations. First, the sample size was relatively small. Besides, the cross-sectional design prevents us from drawing causal inferences between process approach and GMV. Second, the diagnostic criteria of MCI and AD were clinical criteria without the “ATN” framework. Also, considering the heterogeneity of MCI and participants with aMCI are more likely to develop AD, our study may contain this confounding factor. Third, AVLT is an unstructured memory test allowing participants to employ individualized recall strategies. Considerable variability in learning styles may have a significant impact on memory performance and the ability of a test to capture underlying cognitive deficits. Controlled learning paradigms in, for example, Loewenstein–Acevedo Scales of Semantic Interference and Learning (LASSI-L) [62] and Memory Binding Test (MBT) [63, 64], provide a format which the to-be-learned information is organized, minimize the variability of learning strategies and allow the use of retrieval-specific cues to access memory [65]. Future studies can focus on the structured memory tests using controlled learning paradigms to detect the clustering in MCI and AD subjects.

## Conclusion

This study indicated that discriminability, response bias and especially serial clustering of the process approach are promising cognitive biomarkers in neuropsychology assessment of the MCI and AD groups. And discriminability and response bias were correlated with GMV of brain regions related to memory, provided basis of neural mechanisms of process approach. Future studies with larger populations are needed to further explore the potential clinical and scientific utility of the process approach, especially that combined with traditional indices and neuroimaging biomarkers would be beneficial to early detecting and diagnosis of MCI and AD.

### Electronic supplementary material

Below is the link to the electronic supplementary material.

Supplementary Material 1

## Data Availability

Our data were obtained from the Department of Neurology, The First Affiliated Hospital of Anhui Medical University. This study was approved by the Institution Ethics Committee of the First Affiliated Hospital of Anhui Medical University (PJ 2023-12-39). The data supporting the findings of this study are available on request from the corresponding author.
