# Supplementary material for: Process approach as a cognitive biomarker related to gray matter volume in mild cognitive impairment and Alzheimer’s disease
Source: BMC Neurol. 2024 Jun 13;24:199. doi: 10.1186/s12883-024-03711-2 (PMC11170873; doi:10.1186/s12883-024-03711-2)
Supplement: Supplementary file 1 — Supplementary Material 1 [file 12883_2024_3711_MOESM1_ESM.docx]

**Supplemental materials**

**Formulas of the process approach**

We used distribution-free (nonparametric) model as following to measure discriminability (A') [1] and response bias (B"_D_) [2] because of the nonparametric data.

When *H* ≥ *FA*, A' = 0.5 + $\frac{[(H-FA)(1+H-FA)]}{4H(1-FA)}$; When *H* < *FA*, A' = 0.5 – $\frac{[(FA-H)(1+FA-H)]}{4FA(1-H)}$ (1)

B"_D_ = $\frac{[\left( 1-H \right)\left( 1-FA \right)-H\times FA]}{[\left( 1-H \right)\left( 1-FA \right)+H\times FA]}$; When *H* = 1 and *FA* = 0, B"_D_ = 0 (2)

Where *H* = hits rate and *FA* = false-positive rate.

For A', a higher score indicates higher accuracy. No bias is indicated by B"_D_ = 0. Negative numbers represent liberal bias, positive numbers represent conservative bias.

Even if the participants did not use an organizational strategy, the amount of observed clustering would increase as recall increases [3]. In order to control for variations in this chance recall, we considered expectancy formula (EXP_sem_) to correct similar with CVLT-II. An observed semantic clustering (OBS_sem_) occurs anytime when two words in adjacent recall positions are members of the same category. The ListBased Clustering Index of semantic (LBC_sem_) is calculated by subtracting EXP_sem_ from the number of OBS_sem_ for each trial. Semantic clustering was calculated as following and scores of three trials in immediate recall were summed up [3].

LBC_sem_ = OBS_sem_ – EXP_sem_ = OBS_sem_ – $\frac{[(r-1)(m-1)]}{N_{L}-1}$ (3)

Where r = the number of correct words recalled on each trial, m = the number of members of each semantic category on the list, and N_L_ = the total number of words on the list.

Similarly, The ListBased Clustering Index of serial (LBC_ser_) was calculated by dividing the number of observed serial clusters (OBS_ser_) by the number of serial clusters that could be expected to occur by chance (EXP_ser_). Serial clustering was calculated as following and scores of three trials in immediate recall were summed up [3].

LBC_ser_ = OBS_ser_ – EXP_ser_ = OBS_ser_ – $\frac{\left( r-1 \right)}{N_{L}}$ (4)

Where, as above, r = the number of correct words recalled on each trial, and N_L_ = the total number of words on the list. A higher score of LBC_sem_ or LBC_ser_ indicates greater use of learning strategy. Intrusions and repetitions were not included in the calculation.

**REFERENCES**

[1] Snodgrass, J.G., Corwin, J., 1988. Pragmatics of measuring recognition memory: applications to dementia and amnesia. *J Exp Psychol Gen* **117** (1), 34-50. DOI: 10.1037//0096-3445.117.1.34

[2] Donaldson, W., 1992. Measuring recognition memory. *J Exp Psychol Gen* **121** (3), 275-277. DOI: 10.1037//0096-3445.121.3.275

[3] Stricker, J.L., Brown, G.G., Wixted, J., Baldo, J.V., Delis, D.C., 2002. New semantic and serial clustering indices for the California Verbal Learning Test-Second Edition: background, rationale, and formulae. *J Int Neuropsychol Soc* **8** (3), 425-435. DOI: 10.1017/s1355617702813224

**Table S1** ROC analyses of process approach and traditional indices among three groups

|  | AUC | 95%CI | *p* | Cut-off | Sensitivity | Specificity | Youden index |
| --- | --- | --- | --- | --- | --- | --- | --- |
| *HC vs. MCI* |  |  |  |  |  |  |  |
| A' | 0.706 | 0.592-0.807 | **0.001** | ≤0.955 | 0.757 | 0.552 | 0.309 |
| LBC_ser_ | 0.721 | 0.605-0.818 | **<0.001** | ≤1.000 | 0.811 | 0.605 | 0.416 |
| AVLT-IR | 0.742 | 0.628-0.836 | **<0.001** | ≤16 | 0.757 | 0.658 | 0.415 |
| AVLT-SR | 0.713 | 0.597-0.811 | **<0.001** | ≤6 | 0.811 | 0.500 | 0.311 |
| AVLT-LR | 0.669 | 0.550-0.773 | **0.007** | ≤3 | 0.405 | 0.869 | 0.274 |
| AVLT-REC | 0.708 | 0.591-0.807 | **0.001** | ≤20 | 0.578 | 0.726 | 0.304 |
| A'+ AVLT-IR | 0.758 | 0.648-0.868 | **<0.001** | ≤0.432 | 0.811 | 0.631 | 0.442 |
| A'+ AVLT-SR | 0.725 | 0.611-0.840 | **0.001** | ≤0.399 | 0.811 | 0.605 | 0.416 |
| A'+ AVLT-LR | 0.707 | 0.590-0.823 | **0.002** | ≤0.412 | 0.757 | 0.605 | 0.362 |
| A'+ AVLT-REC | 0.709 | 0.592-0.826 | **0.002** | ≤0.699 | 0.378 | 0.948 | 0.326 |
| LBC_ser_+ AVLT-IR | 0.818 | 0.720-0.916 | **<0.001** | ≤0.574 | 0.676 | 0.894 | 0.570 |
| LBC_ser_+ AVLT-SR | 0.802 | 0.700-0.904 | **<0.001** | ≤0.528 | 0.811 | 0.737 | 0.548 |
| LBC_ser_+ AVLT-LR | 0.785 | 0.680-0.890 | **<0.001** | ≤0.478 | 0.811 | 0.684 | 0.495 |
| LBC_ser_+ AVLT-REC | 0.799 | 0.699-0.899 | **<0.001** | ≤0.477 | 0.757 | 0.737 | 0.494 |
| *MCI vs. AD* |  |  |  |  |  |  |  |
| A' | 0.822 | 0.714-0.902 | **<0.001** | ≤0.795 | 0.686 | 0.865 | 0.551 |
| B"_D_ | 0.675 | 0.555-0.781 | **0.006** | ≤-0.200 | 0.600 | 0.703 | 0.303 |
| AVLT-IR | 0.865 | 0.764-0.934 | **<0.001** | ≤10 | 0.743 | 0.838 | 0.581 |
| AVLT-SR | 0.908 | 0.817-0.963 | **<0.001** | ≤2 | 0.886 | 0.811 | 0.697 |
| AVLT-LR | 0.924 | 0.837-0.973 | **<0.001** | ≤1 | 0.943 | 0.865 | 0.808 |
| AVLT-REC | 0.849 | 0.746-0.923 | **<0.001** | ≤16 | 0.743 | 0.838 | 0.581 |
| A'+ AVLT-IR | 0.906 | 0.838-0.974 | **<0.001** | ≤0.386 | 0.914 | 0.811 | 0.725 |
| A'+ AVLT-SR | 0.924 | 0.865-0.982 | **<0.001** | ≤0.574 | 0.857 | 0.865 | 0.722 |
| A'+ AVLT-LR | 0.943 | 0.893-0.993 | **<0.001** | ≤0.423 | 0.943 | 0.865 | 0.808 |
| A'+ AVLT-REC | 0.845 | 0.753-0.936 | **<0.001** | ≤0.499 | 0.743 | 0.811 | 0.554 |
| B"_D_ + AVLT-IR | 0.880 | 0.802-0.958 | **<0.001** | ≤0.479 | 0.857 | 0.784 | 0.641 |
| B"_D_ + AVLT-SR | 0.907 | 0.833-0.981 | **<0.001** | ≤0.568 | 0.886 | 0.838 | 0.724 |
| B"_D_ + AVLT-LR | 0.932 | 0.873-0.991 | **<0.001** | ≤0.504 | 0.943 | 0.865 | 0.808 |
| B"_D_ + AVLT-REC | 0.851 | 0.761-0.941 | **<0.001** | ≤0.589 | 0.743 | 0.838 | 0.581 |
| *HC vs. AD* |  |  |  |  |  |  |  |
| A' | 0.904 | 0.813-0.961 | **<0.001** | ≤0.833 | 0.771 | 0.948 | 0.719 |
| B"_D_ | 0.704 | 0.585-0.805 | **0.001** | ≤-0.615 | 0.543 | 0.842 | 0.385 |
| AVLT-IR | 0.970 | 0.901-0.996 | **<0.001** | ≤12 | 0.857 | 0.948 | 0.805 |
| AVLT-SR | 0.986 | 0.926-1.000 | **<0.001** | ≤3 | 0.971 | 0.922 | 0.893 |
| AVLT-LR | 0.995 | 0.941-1.000 | **<0.001** | ≤2 | 0.971 | 0.974 | 0.945 |
| AVLT-REC | 0.939 | 0.857-0.981 | **<0.001** | ≤19 | 0.943 | 0.816 | 0.759 |
| A'+ AVLT-IR | 0.996 | 0.989-1.000 | **<0.001** | ≤0.157 | 1.000 | 0.947 | 0.947 |
| A'+ AVLT-SR | 0.989 | 0.974-1.000 | **<0.001** | ≤0.296 | 0.971 | 0.921 | 0.892 |
| A'+ AVLT-LR | 0.996 | 0.989-1.000 | **<0.001** | ≤0.157 | 1.000 | 0.947 | 0.947 |
| A'+ AVLT-REC | 0.941 | 0.885-0.998 | **<0.001** | ≤0.335 | 0.943 | 0.816 | 0.759 |
| B"_D_ + AVLT-IR | 0.973 | 0.944-1.000 | **<0.001** | ≤0.266 | 0.943 | 0.868 | 0.811 |
| B"_D_ + AVLT-SR | 0.987 | 0.969-1.000 | **<0.001** | ≤0.371 | 0.971 | 0.921 | 0.892 |
| B"_D_ + AVLT-LR | 0.996 | 0.989-1.000 | **<0.001** | ≤0.472 | 0.971 | 0.974 | 0.945 |
| B"_D_ + AVLT-REC | 0.942 | 0.885-0.998 | **<0.001** | ≤0.407 | 0.886 | 0.894 | 0.780 |

ROC, receiver operating characteristic; AUC, area under the curve; 95%CI, confidence interval of 95%; A', discriminability; B"_D_, response bias; LBC_ser_, ListBased Clustering Index of serial; AVLT-IR, Auditory Verbal Learning Test-Immediately Recall; AVLT-SR, Auditory Verbal Learning Test-short-term delay recall; AVLT-LR, Auditory Verbal Learning Test-long-term delay recall; AVLT-REC, Auditory Verbal Learning Test-recognition; HC, health control; MCI, mild cognitive impairment; AD, Alzheimer’s disease; vs., versus.

**Table S2** AUC comparisons for combined indices and traditional indices

| AUC | 95%CI of difference | *p* |
| --- | --- | --- |
| *HC vs. MCI* |  |  |
| (A' + AVLT-IR) *vs.* AVLT-IR | (-0.037)-0.068 | 0.561 |
| (A' + AVLT-SR) *vs.* AVLT-IR | (-0.084)-0.118 | 0.746 |
| (A' + AVLT-LR) *vs.* AVLT-IR | (-0.072)-0.144 | 0.519 |
| (A' + AVLT-REC) *vs.* AVLT-IR | (-0.090)-0.157 | 0.596 |
| (LBC_ser_ + AVLT-IR) *vs.* AVLT-IR | 0.005-0.147 | **0.036** |
| (LBC_ser_ + AVLT-SR) *vs.* AVLT-IR | (-0.050)-0.170 | 0.287 |
| (LBC_ser_ + AVLT-LR) *vs.* AVLT-IR | (-0.072)-0.157 | 0.465 |
| (LBC_ser_ + AVLT-REC) *vs.* AVLT-IR | (-0.067)-0.181 | 0.367 |
| *MCI vs. AD* |  |  |
| (A' + AVLT-IR) *vs.* AVLT-LR | (-0.064)-0.100 | 0.671 |
| (A' + AVLT-SR) *vs.* AVLT-LR | (-0.034)-0.035 | 0.983 |
| (A' + AVLT-LR) *vs.* AVLT-LR | (-0.007)-0.045 | 0.151 |
| (A' + AVLT-REC) *vs.* AVLT-LR | (-0.013)-0.172 | 0.093 |
| (B"_D_ + AVLT-IR) *vs.* AVLT-LR | (-0.046)-0.133 | 0.337 |
| (B"_D_ + AVLT-SR) *vs.* AVLT-LR | (-0.018)-0.051 | 0.345 |
| (B"_D_ + AVLT-LR) *vs.* AVLT-LR | (-0.012)-0.028 | 0.428 |
| (B"_D_ + AVLT-REC) *vs.* AVLT-LR | (-0.014)-0.160 | 0.101 |
| *HC vs. AD* |  |  |
| (A' + AVLT-IR) *vs.* AVLT-LR | (-0.002)-0.004 | 0.421 |
| (A' + AVLT-SR) *vs.* AVLT-LR | (-0.004)-0.016 | 0.242 |
| (A' + AVLT-LR) *vs.* AVLT-LR | (-0.002)-0.004 | 0.421 |
| (A' + AVLT-REC) *vs.* AVLT-LR | (-0.002)-0.110 | 0.061 |
| (B"_D_ + AVLT-IR) *vs.* AVLT-LR | (-0.004)-0.049 | 0.094 |
| (B"_D_ + AVLT-SR) *vs.* AVLT-LR | (-0.005)-0.022 | 0.220 |
| (B"_D_ + AVLT-LR) *vs.* AVLT-LR | (-0.003)-0.006 | 0.629 |
| (B"_D_ + AVLT-REC) *vs.* AVLT-LR | (-0.003)-0.110 | 0.064 |

AUC, area under the curve; 95%CI, confidence interval of 95%; A', discriminability; B"_D_, response bias; LBC_ser_, ListBased Clustering Index of serial; AVLT-IR, Auditory Verbal Learning Test-Immediately Recall; AVLT-SR, Auditory Verbal Learning Test-short-term delay recall; AVLT-LR, Auditory Verbal Learning Test-long-term delay recall; AVLT-REC, Auditory Verbal Learning Test-recognition; HC, health control; MCI, mild cognitive impairment; AD, Alzheimer’s disease; vs., versus.

**Table S3** Significant clusters of comparison of HC, MCI and AD groups

| Cluster | Brain region | Cluster size | MNI coordinate | | | *F* value |
| --- | --- | --- | --- | --- | --- | --- |
|  | (AAL) | (voxels) | X | Y | Z |  |
| Cluster 1 | MTG.R | 2547 | 55.5 | -43.5 | 46.5 | 25.830 |
| Cluster 2 | HIP.R | 2495 | 28.5 | -27 | -12 | 46.836 |
| Cluster 3 | PHG.L | 2200 | -24 | -34.5 | -7.5 | 48.914 |
| Cluster 4 | MCG.R | 1013 | 9 | -49.5 | 37.5 | 25.501 |
| Cluster 5 | ITG.R | 987 | 48 | -46.5 | -22.5 | 25.140 |
| Cluster 6 | THA.R | 523 | 1.5 | -10.5 | 12 | 26.419 |
| Cluster 7 | MTG.L | 520 | -55.5 | -9 | -10.5 | 20.346 |

AAL, anatomical automatic labeling; MNI, Montreal Neurological Institute; HC, health control; MCI, mild cognitive impairment; AD, Alzheimer’s disease; MTG.R, right middle temporal gyrus; HIP.R, right hippocampus; PHG.L, left parahippocampal gyrus; MCG.R, right middle cingulate gyrus; ITG.R, right inferior temporal gyrus; THA.R, right thalamus; MTG.L, left middle temporal gyrus.
